# Oxidative Stress in Metabolic and Endocrine Diseases: Basic and Translational Aspects

**DOI:** 10.3390/ijms23084346

**Published:** 2022-04-14

**Authors:** Antonio Mancini, Andrea Silvestrini

**Affiliations:** 1 Dipartimento di Medicina e Chirurgia Traslazionale, Università Cattolica del Sacro Cuore, 00168 Rome, Italy; antonio.mancini@unicatt.it; 2 Dipartimento di Scienze Biotecnologiche di Base, Cliniche Intensivologiche e Perioperatorie, Università Cattolica del Sacro Cuore, 00168 Rome, Italy

The aim of this Special Issue is to highlight oxidative stress (OS) as a mechanism underlying a major risk factor for several human diseases. Interestingly, the complex inter-relationships between hormones, oxidative status, and inflammation are still not completely understood since reciprocal and opposite influences can be exerted between these pathogenic factors in endocrine disease. Moreover, biochemical/biological data and their application in clinical trials are still distant and require a translational approach. The concept of what constitutes a hormone itself is changing, including virtually all organs and systems. Thus, hormones also appear to be key modulators of antioxidant defenses [1,2]. Therefore, we aimed to publish a Special Issue that covers a large area, from biological to translational studies, in the field of endocrine and metabolic disorders. Articles published in this Special Issue cover three main topics: obesity and metabolic syndrome with risk factors associated with their complications; endocrinology of reproduction, with a special focus on male infertility; oncology.

The first research article by Mohammed et al. [3] investigated obesity through an interesting experimental model represented by a high-fat diet (HFD) in mice. Obesity is not only a risk factor for diabetes and other components of metabolic syndrome but is a disease state itself, associated with low-grade inflammation and insulin resistance, with a complex cascade of intracellular signaling. In this article, molecular mechanisms affecting the retinal microvascular structure and function are evaluated. The role of the thioredoxin-interacting protein (TXNIP), which is linked to retinal NOD-like receptor protein-inflammasome, activated by HFD, has been investigated by comparing the effects of HFD in wild-type and TXNIP knock-out mice, using both cultured endothelial cells and ex vivo leukocytes. Early retinal leukostasis and microvascular dysfunctions were shown in wild-type but not in TXNIP knock-out models. Whether this model can be generalized to better understand the role of hyperglycemia, fatty acids, or insulin resistance itself in inducing microvascular complications of obesity and metabolic syndrome remain to be established.

Non-alcoholic fatty liver disease (NAFLD) is a chronic condition related to insulin resistance and therefore proposed as a component of metabolic syndrome; it covers a large spectrum of situations, ranging from steatosis to steatohepatitis, with a possible progressive evolution toward cirrhosis and hepatocarcinoma. There is still debate on the mechanisms underlying this differential clinical course; both OS and innate/adaptive immune response could contribute. The review article of Dallio et al. [4] describes their reciprocal influence in a vicious circle, leading to NAFLD progression. In such a situation, both mitochondrial and endoplasmic reticulum contribute to OS; on the other hand, redox balance profoundly affects the immune system. OS is considered a trigger for adaptive immunity via the formation of products derived by lipid peroxidation and altogether named “oxidation-specific epitopes” (OSEs); however, the contribution of innate immunity, with the production of cytokines and new reactive species, has been described. The interesting concept of “trained immunity” (TI), including the totality of immunological processes that involve non-specific immune cells, such as monocytes and natural killers, after a second antigenic contact, represents an innovative frontier in medicine; the therapeutic consequences are very fascinating. Due to the well-documented involvement of immune mechanisms in hepatocellular carcinoma, the administration of immune checkpoint inhibitors represents a new scenario that requires further validation.

Antioxidants have been proposed to counteract progressive damage to insulin resistance. We have previously reported that a diet enriched with natural antioxidants can have a beneficial effect, including promoting the therapeutic efficacy of metformin [5]. Accordingly, the pleiotropic activities of the antioxidant resveratrol are described in the review by Mongioì et al. [6]. Resveratrol, a polyphenol extracted from the roots of white hellebore, has been shown to have antioxidant and anticancer properties. It modulates apoptosis factors, protects against DNA damage, and has anti-inflammatory actions. Moreover, neuroprotective, anti-infectious and antiaging properties have been attributed to resveratrol. The article analyzes in detail the mechanisms of resveratrol’s effects in obesity. The activation of deacetylase Sirtuin-1 seems to be a key phenomenon, but it also modulates AMP-kinase. The effects of resveratrol on adipocytes are also described and seem to be in part direct, via the modulation of gene expression, and in part indirect, mediated by SIRT1 activation; the latter is also involved in PGC-1α activation and the consequent metabolic effects, supporting thermogenesis in muscle and brown adipose tissue. Despite biochemical evidence and preclinical studies, the effects in humans are still poorly understood. The article shows the studies performed on human obese patients; however, most studies have been conducted in small groups and with different schedules of doses and times of administration. The unique large study (in more than 100 patients) did not show variations in cardio-metabolic risk factors and liver fat content, while more recent studies suggest that the amelioration of some parameters can be obtained if the treatment is combined with a diet and exercise program. Thus, the authors conclude that more studies in a larger population can establish resveratrol’s real long-term effects.

Similar conclusions are suggested in the review article by Perez-Torres et al. [7], who present characteristics and mechanism of action of numerous plant natural antioxidant compounds, including polyphenols, carotenoids, capsaicinoids and casinoids, isothyocianates, catechins and vitamins. All these compounds can exert scavenger action on ROS and anti-inflammatory activities (preventing the activation of NFkB), reducing the expression of genes involved in inflammations.

OS is an extensively studied mechanism also involved in male infertility. Even though radicals can have a physiological positive role, their excess has been documented in approximately 30–80% of infertile men; in some cases, an association with specific etiologies has been described, such as varicocele, low urinary tract infections, exogenous toxins such as smoke and so on, many situations remain unexplained, and OS alone could be the cause of infertility. In fact, OS can damage sperm membrane but also induce sperm DNA fragmentation. More recently, proteomics analysis appears to be a candidate as an OS biomarker in seminal fluid due to the number of proteins present both in sperm cells and in the seminal plasma due to prostatic and vesicular secretion. The review article by Cannarella et al. [8] summarized and discussed the proteomics changes related to OS. Nine proteins were overexpressed in patients with OS; instead, 23 proteins were differentially expressed in specific conditions: varicocele, male accessory gland infections, cigarette smoke, and obesity. Interestingly, these specific patterns do not overlap each other, suggesting their possible role as diagnostic and prognostic markers.

Sperm DNA fragmentation is an alteration linked to OS and a possible cause of impaired fertilization, embryo quality, implantation, and the course of pregnancy, all biological processes that require sperm DNA integrity. Agarwal et al. [9], in their review article, clearly show mechanisms underlying DNA fragmentation, which can be summarized as altered chromatin condensation (which is related to the replacement of protamine to histone in the nucleosome), abortive apoptosis of damaged cells, escaping from clearing action of Sertoli cells, and finally, lipid peroxidation and formation of 8-OH-derivatives of guanosine and deoxyguanosine. Among different methods employed in clinical practice to explore this phenomenon, in the authors’ opinion, the Comet assay can better differentiate two kinds of damages (single-and double-strain breaks). They present evidence of the impact of DNA fragmentation on outcomes of assisted reproductive techniques, including fertilization and implantation rates, miscarriage and pregnancy rate, and life birth rate. Interestingly, double-strand breaks appear to be more harmful; therefore, the authors underline the importance to evaluate this datum before sperm selection in techniques such as ICSI (intracytoplasmic sperm injection).

These two areas (i.e., metabolism and infertility) are very likely not completely independent. Hyperhomocysteinemia (HH) is a biochemical phenomenon related to endothelial dysfunction and the onset/progression of atherosclerosis. Among many consequences in the cardiovascular system, Salvio et al. [10] comprehensively summarized the relationships, both from a biochemical and clinical point of view, in erectile dysfunction. It is largely agreed upon that HH can alter many properties of the endothelium via OS. The release of nitric oxide by the endothelium in corpora cavernosa is critical for full erection; studies in animals and in humans show that HH is coupled with decreased NO production, but a direct pro-oxidant mechanism has been shown and reversed by enzymatic antioxidants. Evidence for HH as a risk factor is also reported in humans; the authors report the basis for clinical trials, which are promising but still to be confirmed.

Interestingly, the topic of OS is also crucial in female reproduction. Polycystic Ovary Syndrome (PCOS) is a gynecological endocrine disorder, in which OS can be the basis for the clinical and biochemical characteristics (hyperandrogenism, anovulation, and insulin resistance). In the review article by Mancini et al. [11], they suggested a paradigm that is in some ways opposite to the usual interpretation, which considers insulin resistance a consequence of OS. In fact, OS itself can induce and/or worsen all main features of PCOS: insulin resistance, obesity, hyperandrogenism, follicular apoptosis, and infertility. Moreover, low-grade inflammation (LGI), in a mutual reinforcing action with OS, seems to be crucial in the pathogenesis of PCOS, both in normal weight and obese patients. Both could be induced by the interaction between genetic background and lifestyle elements, while hormonal events (e.g., hyperandrogenism) can be factors inducing or amplifying the OS-LGI status. In addition, considering the heterogeneity of the PCOS population, targeted therapies focused on pathophysiological mechanisms should be considered, even if scientific evidences of this approach are still lacking.

The next research article by Wang et al. [12] opens the question of antioxidant effects on cancer cell metabolism. The model is that of MCF7 breast cancer cells, and the redox status in this case is Coenzyme Q (CoQ) depletion, a key molecule with well-known bioenergetics and antioxidant properties. CoQ depletion was obtained by competitive inhibition of the final step of its biosynthetic machinery. In this case, cancer cells were shown to adapt to energetic/oxidative stress: in CoQ depleted cells, the glycolysis was upregulated together with an increase in glucose consumption, overexpression of glucose transporters (GLUT-1 and GLUT-3), and activation of glycolytic enzymes, such as pyruvate-kinase. The lactate secretion rate was reduced, suggesting a redirection of pyruvate metabolism in the anabolic pathway; finally, a different pattern of expression of enzymes involved in glutamine metabolism and the tricarboxylic acid cycle was observed. Again, these data, other than their physiopathological importance, can offer the basis for an approach targeted at breast cancer.

Stimulatingly, all these models can be extended to other fields of medical practice (e.g., dermatology), such as vitiligo. Due to OS involvement in skin diseases and the capacity of estrogens to counteract OS, Yamamoto et al. [13], in their research article, investigated the antioxidant effects of local estrogens in epidermal cells. The authors report that the main biosynthetic enzyme—17B-hydroxysteroid-dehydrogenase (HSD17β1)—was expressed in keratinocytes, while the most dominant receptor—G-coupled estrogen receptor 1—was expressed in melanocytes. Keratocyte-derived estradiol is able to counteract H_2_O_2_-induced cell death; on the other hand, reduced levels of HSD17β1 are found in the epidermis of skin obtained by vitiligo patients.

Finally, due to the heterogeneity of the articles included in the Special Issue—and perhaps the very specific topics that could be of interest for researchers involved in related fields—it is clear that investigations concerning OS transversally share a great interest in biological and clinical aspects of medicine. Overall, the topic of OS in endocrine and metabolic diseases (including examples such as pituitary, thyroid, and adrenal diseases) [1] already has many aspects to be deepened and could attract the attention and interest of the scientific community. A great gap between basic/translational and clinical studies has yet to be tightened. Thus, randomized and large controlled studies in humans to further contribute to future research are needed. Nonetheless, the topic of OS is provoking, and we think that the effort to correlate biochemical data with personalized health can be further pursued.
